# Prediction of implant failure risk due to periprosthetic femoral fracture after primary elective total hip arthroplasty: a simplified and validated model based on 154,519 total hip arthroplasties from the Swedish Arthroplasty Register

**DOI:** 10.1302/2046-3758.141.BJR-2024-0134.R1

**Published:** 2025-01-24

**Authors:** M. Abdulhadi Alagha, Justin Cobb, Alexander D. Liddle, Henrik Malchau, Ola Rolfson, Maziar Mohaddes

**Affiliations:** 1 MSk Lab, Department of Surgery and Cancer, Faculty of Medicine, Imperial College London, London, UK; 2 Data Science Institute, London School of Economics and Political Science, London, UK; 3 Harvard Medical School, Boston, Massachusetts, USA; 4 Department of Orthopaedics, Institute of Clinical Sciences, University of Gothenburg, Gothenburg, Sweden

**Keywords:** Machine learning, Total hip arthroplasty, Periprosthetic fracture, periprosthetic femoral fractures, implant failure, revision surgery, Anesthesiologists, SAR, femoral heads, cementless fixation, hip resurfacing arthroplasty, Arthroplasty registries, idiopathic necrosis

## Abstract

**Aims:**

While cementless fixation offers potential advantages over cemented fixation, such as a shorter operating time, concerns linger over its higher cost and increased risk of periprosthetic fractures. If the risk of fracture can be forecasted, it would aid the shared decision-making process related to cementless stems. Our study aimed to develop and validate predictive models of periprosthetic femoral fracture (PPFF) necessitating revision and reoperation after elective total hip arthroplasty (THA).

**Methods:**

We included 154,519 primary elective THAs from the Swedish Arthroplasty Register (SAR), encompassing 21 patient-, surgical-, and implant-specific features, for model derivation and validation in predicting 30-day, 60-day, 90-day, and one-year revision and reoperation due to PPFF. Model performance was tested using the area under the curve (AUC), and feature importance was identified in the best-performing algorithm.

**Results:**

The Lasso regression excelled in predicting 30-day revisions (area under the receiver operating characteristic curve (AUC) = 0.85), while the Gradient Boosting Machine (GBM) model outperformed other models by a slight margin for all remaining endpoints (AUC range: 0.79 to 0.86). Predictive factors for revision and reoperation were identified, with patient features such as increasing age, higher American Society of Anesthesiologists grade (> III), and World Health Organization obesity classes II to III associated with elevated risks. A preoperative diagnosis of idiopathic necrosis increased revision risk. Concerning implant design, factors such as cementless femoral fixation, reverse-hybrid fixation, hip resurfacing, and small (< 35 mm) or large (> 52 mm) femoral heads increased both revision and reoperation risks.

**Conclusion:**

This is the first study to develop machine-learning models to forecast the risk of PPFF necessitating secondary surgery. Future studies are required to externally validate our algorithm and assess its applicability in clinical practice.

Cite this article: *Bone Joint Res* 2025;14(1):46–57.

## Article focus

- The aim of this study was to develop machine learning (ML)-based risk prediction models and compare their predictive ability to traditional statistical linear models using large datasets from the Swedish Arthroplasty Register.
- The outcome measure was to predict the risks of reoperation and revision due to periprosthetic femoral fracture (PPFF) at multiple timepoints ranging from 30 days to one year postoperatively.

## Key messages

- This study has demonstrated that ML-based predictive models for revision and reoperation due to PPFFs after primary hip arthroplasty achieved a relatively higher level of accuracy, as determined by the area under the curve (AUC), compared to the logistic regression linear model.
- Our findings identified several patient characteristics and implant-specific features to be associated with revision and reoperation after total hip arthroplasty (THA) due to PPFF at each timepoint. For the early (30 to 90 days) revision surgery, these included patient features (increasing age and comorbidities), preoperative diagnosis (idiopathic necrosis), and implant design (cementless femoral fixation, hybrid fixation, hip resurfacing, and small (< 25 mm) or large (> 45 mm) femoral heads).

## Strengths and limitations

- This is the first study to develop ML models to forecast the risk of PPFF necessitating revision surgery.
- The retrospective analysis of prospectively collected observational data in a single national joint registry, as well as the limitations concerning the use of revision/reoperation as endpoint outcomes, may hinder the clinical applicability to other settings.
- We compared our models using the AUC metric, which is regarded as a gold standard in evaluating classifier performance, however it neglects to address the balance of true positives and true negatives, and the potential bias introduced by the low incidence of reoperation may affect the accuracy and generalizability of the predictive model.

## Introduction

Total hip arthroplasties (THAs) are effective orthopaedic surgeries, typically indicated in patients with end-stage osteoarthritis.^1^ Cementless fixation for the femoral stem gained popularity in the late 1980s and are widely used in Australia, Canada, England and Wales, and Italy.^2^ However, there remains controversy about whether cemented or cementless fixation provides superior outcomes. Cementless fixation can be more expensive than cemented, but this is offset by a shorter operating time.^3^ Cementless fixation also has a higher risk of periprosthetic fracture (PPF) owing to the press-fit required to achieve primary stability of the stem,^4-6^ with intraoperative periprosthetic femoral fracture (PPFF) 14-times more likely to occur postoperatively with cementless implants.^4^ If the risk of PPF can be reduced, it will improve the outcome of cementless stems.

Examination of the Swedish Arthroplasty Register’s (SAR) 2022 annual report reveals that PPF is the third leading cause for first-time revision hip arthroplasty (13.2%) following aseptic loosening (44%) and infection (24.5%).^7^ A previous study conducted by the SAR between 1992 and 2007 found a higher risk of PPFF requiring revision within two years following primary THA in cementless fixation compared to cemented fixation (17% vs 6%; adjusted relative risk (RR) 8.0 (4.5 to 14)).^8^ The annual report of the National Joint Registry of England and Wales in 2022 yielded similar findings with PPF, the four predominant causes for revision being aseptic loosening (24.6%), dislocation/subluxation (17.4%), PPF (15.7%), and infection (15.2%).^9^ The annual report shows a higher incidence of revisions per 1,000 prosthesis-years for PPFF following primary hip arthroplasty in cementless fixation (0.67%) than cemented fixation (0.53%), with hybrid fixation (0.86%) and reverse hybrid fixation (0.66%) also displaying relatively elevated rates.^9^ According to the Australian Orthopaedic Association National Joint Arthroplasty Registry’s (AOANJRR) annual report of 2022,^10^ PPF is the third leading reason for revision following primary THA (21.8%), after infection (22.7%) and prosthesis dislocation/instability (22.0%). AOANJRR reports no difference in revision rate between cemented and hybrid fixations for all age groups, while cementless fixation has a higher revision risk following primary THA compared to hybrid and cemented fixation in patients aged 75 years and above, and a time-dependent increased revision risk for the 55 to 64 years (first month postoperatively) and 65 to 74 years (1.5 years postoperatively) age groups. Machine-learning (ML) predictive models offer an alternative which has the potential to better forecast future events, while accounting for a large number of predictor variables and their potentially complex and non-linear interactions.^11^ Several studies have demonstrated the superior predictive accuracy of ML in healthcare over traditional risk stratification approaches.^12-17^ Although relatively rare, the occurrence of postoperative PPFs is on the rise and presents significant challenges in the context of primary hip arthroplasty. These complications are linked to a heightened risk of negative health outcomes, increased mortality rates, and significant financial burdens, and also pose technical difficulties for surgeons.^18,19^

Given this context, it is crucial to distinguish between intraoperative PPFs, postoperative PPFs, and missed intraoperative fractures that progress to completion. Registry-recorded outcomes are primarily postoperative, as intraoperative fractures are managed during the primary procedure and therefore do not qualify as revisions or reoperations.

The aim of this study was to develop ML-based risk prediction models and compare their predictive ability to traditional statistical linear models using large datasets from the SAR. The outcome measure was to predict the risks of reoperation and revision due to PPFF at multiple timepoints ranging from 30 days to one year postoperatively.

## Methods

The SAR dataset (n = 442,546) was used for model derivation and internal validation for predicting revision and reoperation outcomes due to PPFF between 2008 and 2018. The study focused on adult patients undergoing elective primary hip arthroplasty, and we thus excluded cases with a diagnosis of trauma, tumour, and nonosteoarthritic degenerative conditions.

Data related to primary hip arthroplasty are collected for all nationwide public and private hospitals in Sweden and report to the register with an estimate completeness of 98%.^20^ Our inclusion period started in 2008 due to the absence of systematic inclusion of BMI and American Society of Anesthesiologists (ASA)^21^ grade in earlier records. The extract contained patients’ demographic, operative, and component-level data, and the SAR is periodically synchronized with The Tax Office and National Patient Register (NPR), which provides information on mortality, causes, and time to event. We examined all variables for missing data, and records with missing values exceeding 60% were excluded under the assumption that the missing data occurred at random (n = 154,595 THAs; Table I). Observations characterized by the absence of recorded PPFFs and those with periprosthetic acetabular fractures were excluded, resulting in a final dataset of 154,519 THAs. The cleaned dataset included 21 variables (Supplementary Table i) : six patient variables (age, sex, BMI, ASA grade, side of operation, and the type of hospital), four surgical variables (diagnosis group, incision type, prosthesis group, and cement type), and 11 implant-specific variables which were each subdivided into six cup variables and five femoral variables (fixation, resurfacing, articulation, modular or monoblock, and implant size; in addition to the sixth cup-related variable ‘modification’). Potential correlations among predictors were examined, and any highly correlated variables were excluded to address multicollinearity and ensure model robustness. Each variable is described in more detail in Supplementary Table ii.

**Table I.** Baseline characteristics of the study population in the Swedish Hip Arthroplasty Register after data cleaning.

Characteristic	Value
Number of operations	154,595
Mean age at surgery, yrs (SD)	67.9 (10.6)
Male sex, n (%)	66,868 (43.3)
**BMI, n (%)**	
Underweight	1,191 (0.8)
Normal	48,037 (31.1)
Overweight	66,408 (43.0)
**WHO obesity class, n (%)**	
I	29,757 (19.2)
II	7,647 (4.9)
III	1,555 (1.0)
**ASA grade, n (%)**	
I	36,826 (23.8)
II	92,076 (59.6)
III	25,693 (16.6)
Right side, n (%)	70,000 (45.3)
**Unit type, n (%)**	
University hospital	12,244 (7.9)
County hospital	47,052 (30.4)
Rural hospital	60,254 (39.0)
Private hospital	35,045 (22.7)
**Preoperative diagnosis, n (%)**	
Primary arthrosis	141,412 (91.5)
Inflammatory	1,962 (1.3)
Childhood diagnosis	3,256 (2.1)
Idiopathic necrosis	3,741 (2.4)
Secondary arthrosis	4,097 (2.7)
Other	127 (0.1)
**Incision type, n (%)**	
Posterior	83,146 (53.8)
Direct lateral	70,924 (45.9)
Direct anterior	324 (0.2)
Trochanteric	173 (0.1)
Other	12 (0.0)
**Prosthesis group, n (%)**	
Cemented	95,490 (61.8)
Cementless	33,479 (21.7)
Hybrid	5,030 (3.3)
Reverse hybrid	19,528 (12.6)
Resurfacing	1,068 (0.7)
**Cement type, n (%)**	
High viscosity with antibiotic	55,195 (35.7)
Low viscosity with antibiotic	876 (0.6)
High viscosity without antibiotic	58,915 (38.1)
Low viscosity without antibiotic	11 (0.0)
Cementless, hybrid or resurfacing	39,577 (25.6)
**Cup fixation, n (%)**	
Cemented	115,020 (74.4)
Cementless	39,575 (25.6)
Cup resurfacing	1,528 (1.0)
Cup modularity (monoblock)	115,818 (74.9)
**Cup articulation, n (%)**	
Metal (standard)	235 (0.2)
Metal (resurfacing)	1,531 (1.0)
Ceramic	637 (0.4)
Dual-mobility (monoblock)	1,278 (0.8)
Dual-mobility (modular)	5 (0.0)
Poly (standard)	44,116 (28.5)
Poly (cross-linked)	106,628 (69.0)
Unclear	165 (0.1)
**Cup modification, n (%)**	
Standard	100,836 (65.2)
Lipped	12,319 (8.0)
Dual articular	1,146 (0.7)
Constrained	10 (0.0)
Unclear	40,284 (26.1)
Mean cup inner diameter, mm (SD)	31.49 (3.05)
**Stem fixation, n (%)**	
Cemented	101,585 (65.7)
Cementless	53,010 (34.3)
Stem resurfacing	1,068 (0.7)
Stem modularity (modular)	153,466 (99.3)
**Stem articulation, n (%)**	
Metal	127,730 (82.6)
Ceramic	25,766 (16.7)
Unclear	1,099 (0.7)
Mean femoral head size, mm (SD)	31.49 (3.03)

ASA, American Society of Anesthesiologists; WHO, World Health Organization.

This study is in two parts: the first part uses the SAR dataset to develop and internally validate the algorithms in predicting revision and reoperation outcomes due to PPFF, followed by a comparison of the key features driving the predictions for the top performing ML model and the traditional statistical model, logistic regression.

### Outcome of interest and definition

The primary outcome measure in the SAR dataset was PPFF risk leading to either a revision or reoperation procedure within 30 days, 60 days, 90 days, and one year postoperatively. Reoperation was defined as any additional surgery on the hip, regardless of the actions taken to any of the implant components (replaced, extracted, or left untouched), whereas revision is defined as a reoperation where at least one component is exchanged, extracted from, or added to the prosthesis. PPF necessitating revision surgery is a specific subset of revision outcomes within the SAR, where the prosthesis undergoes modification by exchanging, extracting, or adding at least one component due to the occurrence of a PPF.

### Model development, training, and validation

Six ML algorithms were developed, namely random forest (RF), gradient boosting machine (GBM), penalized logistic regression (with Lasso and Ridge penalty), and classification tree (with and without pruning), and then compared to traditional statistical model logistic regression to predict the occurrence of intra-/postoperative PPFF necessitating a secondary procedure. The SAR cohort of 154,519 observations was used for model development and internal validation. The data extract was randomly split into a training dataset (n = 123,615, 80% of the patients) and an internal validation cohort (n = 30,904, 20% of the patients). The training cohort was used to train the ML models and to adjust their hyperparameters via cross-validation, whereas the validation cohort was used to assess the models’ performance on unseen data. Model performance was evaluated and compared in terms of area under the receiver operating characteristic curve (AUC) in each dataset. The top-performing ML model was used to identify the key features driving its performance.

### Statistical analysis

Descriptive statistics, including mean and percentage, were used to describe the rates of implant fracture across various age and sex groups as well as per year in the SAR datasets. Kaplan-Meier (KM) non-parametric estimates were employed to describe the rates of PPFF leading to revision or reoperation procedures across various age and sex groups in the SAR dataset.^22^ All mathematical modelling was carried out using R statistical computing environment version 4.3.0 (R Foundation for Statistical Computing, Austria). R packages ‘survival’ (version 3.5.5), ‘gbm’ (version 2.1.8.1), ‘glmnet’ (version 4.1.7), ‘tree’ (version 1.0.43), ‘rpart’ (version 4.7.19), and ‘randomForest’ (version 4.7.1.1) were used for survival analysis.

## Results

### Summary statistics of outcomes

Descriptive figures related to the SAR PPFF outcomes are presented in Table II. The one-year rates for revision and reoperation were 0.17% and 0.23%, respectively.

**Table II.** Analysis of study outcome rates in the Swedish Arthroplasty Register dataset.

Outcome	Time from primary operation	Derivation cohort, n (%) (n = 154,519)
Revision due to periprosthetic femoral fractures	30 days	120 (0.07)
	60 days	169 (0.10)
	90 days	196 (0.12)
	1 year	266 (0.17)
Reoperation due to periprosthetic femoral fractures	30 days	139 (0.08)
	60 days	202 (0.13)
	90 days	239 (0.15)
	1 year	369 (0.23)

Table III provides Kaplan-Meier estimates with 95% CIs for the percentage of patients experiencing revision due to PPFF at different time intervals following primary elective hip arthroplasty, stratified by sex and age groups. Overall, one-year revision rates due to fracture ranged from 0.00% to 0.23% with no apparent discrepancy between sex groups.

**Table III.** Summary and Kaplan-Meier estimates for the cumulative incidence of revision outcomes due to periprosthetic femoral fractures at each timepoint by age and sex.

Patients	N total	30-day revision		60-day revision		90-day revision		One-year revision	
		**N**	**KM estimate, %** **(95% CI)**	**N**	**KM estimate, %** **(95 CI)**	**N**	**KM estimate, %** **(95 CI)**	**N**	**KM estimate, %** **(95% CI)**
All patients	154,519	120	0.05 (0.02 to 0.10)	169	0.08 (0.03 to 0.13)	196	0.10 (0.05 to 0.15)	266	0.13 (0.07 to 0.19)
**Male**									
All	66,830	50	0.05 (0.02 to 0.10)	68	0.08 (0.03 to 0.13)	84	0.10 (0.05 to 0.16)	130	0.15 (0.08 to 0.22)
18 to 34 yrs	393	0	0.00 (0.00 to 0.00)	0	0.00 (0.00 to 0.00)	0	0.00 (0.00 to 0.00)	0	0.00 (0.00 to 0.00)
35 to 49 yrs	4,244	1	0.02 (0.00 to 0.07)	2	0.05 (0.00 to 0.11)	3	0.07 (0.00 to 0.15)	5	0.12 (0.02 to 0.23)
50 to 59 yrs	11,264	12	0.11 (0.05 to 0.17)	18	0.16 (0.09 to 0.24)	21	0.19 (0.11 to 0.27)	24	0.22 (0.13 to 0.31)
60 to 69 yrs	22,783	15	0.07 (0.03 to 0.10)	21	0.09 (0.05 to 0.13)	26	0.12 (0.07 to 0.16)	49	0.23 (0.16 to 0.29)
70 to 79 yrs	21,276	17	0.08 (0.04 to 0.12)	19	0.09 (0.05 to 0.13)	24	0.11 (0.07 to 0.16)	41	0.20 (0.14 to 0.26)
≥ 80 yrs	6,870	5	0.07 (0.01 to 0.14)	8	0.12 (0.04 to 0.20)	10	0.15 (0.06 to 0.24)	11	0.17 (0.07 to 0.26)
**Female**									
All	87,689	70	0.06 (0.02 to 0.10)	101	0.09 (0.04 to 0.14)	112	0.10 (0.05 to 0.15)	136	0.12 (0.06 to 0.17)
18 to 34 yrs	415	0	0.00 (0.00 to 0.00)	0	0.00 (0.00 to 0.00)	0	0.00 (0.00 to 0.00)	0	0.00 (0.00 to 0.00)
35 to 49 yrs	3,325	2	0.06 (0.00 to 0.14)	3	0.09 (0.00 to 0.19)	3	0.09 (0.00 to 0.19)	3	0.09 (0.00 to 0.19)
50 to 59 yrs	10,903	12	0.11 (0.05 to 0.17)	19	0.18 (0.10 to 0.26)	20	0.19 (0.10 to 0.27)	21	0.20 (0.11 to 0.28)
60 to 69 yrs	27,548	32	0.12 (0.08 to 0.16)	40	0.15 (0.10 to 0.19)	42	0.15 (0.11 to 0.20)	52	0.19 (0.14 to 0.24)
70 to 79 yrs	32,267	21	0.07 (0.04 to 0.09)	33	0.10 (0.07 to 0.14)	39	0.12 (0.08 to 0.16)	46	0.15 (0.10 to 0.19)
≥ 80 yrs	13,231	3	0.02 (0.00 to 0.05)	6	0.05 (0.01 to 0.08)	8	0.06 (0.02 to 0.10)	14	0.11 (0.05 to 0.17)

KM, Kaplan-Meier.

Supplementary Table iii highlights the temporal variations in Kaplan-Meier estimates for revision due to PPFF over successive years between 2008 and 2018. No statistically significant differences were detected throughout all the years.

Kaplan-Meier estimates, stratified by age, sex, and year, for the cumulative incidence of reoperation due to PPFFs at four intervals are presented in Table IV and Supplementary Table iv. No observed differences were found between males and females, while the incidence of fractures necessitating reoperation was highest in the 50- to 69-year age groups. No statistically significant differences were detected throughout the years.

**Table IV.** Summary and Kaplan-Meier (KM) estimates for the cumulative incidence of reoperation outcomes due to periprosthetic femoral fractures at each timepoint by age and sex.

All patients	N total	30-day reoperation		60-day reoperation		90-day reoperation		1-year reoperation	
		**N**	**KM estimate, %** **(95% CI)**	**N**	**KM estimate, %** **(95% CI)**	**N**	**KM estimate, %** **(95% CI)**	**N**	**KM estimate, %** **(95% CI)**
	154,519	139	0.07 (0.02 to 0.11)	202	0.10 (0.05 to 0.16)	239	0.12 (0.06 to 0.19)	369	0.20 (0.12 to 0.28)
**Male patients**									
All	66,830	53	0.06 (0.02 to 0.10)	76	0.09 (0.04 to 0.14)	97	0.12 (0.06 to 0.19)	166	0.21 (0.12 to 0.29)
18 to 34 yrs	393	0	0.00 (0.00 to 0.00)	0	0.00 (0.00 to 0.00)	0	0.00 (0.00 to 0.00)	0	0.00 (0.00 to 0.00)
35 to 49 yrs	4,244	1	0.02 (0.00 to 0.07)	2	0.05 (0.00 to 0.11)	4	0.10 (0.00 to 0.19)	8	0.20 (0.06 to 0.33)
50 to 59 yrs	11,264	13	0.12 (0.05 to 0.18)	21	0.19 (0.11 to 0.27)	25	0.23 (0.14 to 0.32)	28	0.26 (0.16 to 0.35)
60 to 69 yrs	22,783	16	0.07 (0.04 to 0.11)	23	0.10 (0.06 to 0.14)	28	0.13 (0.08 to 0.17)	59	0.27 (0.20 to 0.34)
70 to 79 yrs	21,276	18	0.09 (0.05 to 0.12)	21	0.10 (0.06 to 0.14)	28	0.13 (0.08 to 0.18)	54	0.27 (0.20 to 0.34)
≥ 80 yrs	6,870	5	0.07 (0.01 to 0.14)	9	0.13 (0.05 to 0.22)	12	0.18 (0.08 to 0.28)	17	0.26 (0.14 to 0.38)
**Female patients**									
All	87,689	86	0.08 (0.03 to 0.12)	126	0.12 (0.06 to 0.18)	142	0.13 (0.07 to 0.19)	203	0.20 (0.12 to 0.28)
18 to 34 yrs	415	0	0.00 (0.00 to 0.00)	0	0.00 (0.00 to 0.00)	0	0.00 (0.00 to 0.00)	0	0.00 (0.00 to 0.00)
35 to 49 yrs	3,325	3	0.09 (0.00 to 0.19)	5	0.15 (0.02 to 0.28)	5	0.15 (0.02 to 0.28)	9	0.28 (0.10 to 0.46)
50 to 59 yrs	10,903	13	0.12 (0.05 to 0.18)	20	0.19 (0.10 to 0.27)	21	0.19 (0.11 to 0.28)	23	0.21 (0.13 to 0.30)
60 to 69 yrs	27,548	37	0.13 (0.09 to 0.18)	49	0.18 (0.13 to 0.23)	52	0.19 (0.14 to 0.24)	71	0.26 (0.20 to 0.33)
70 to 79 yrs	32,267	24	0.07 (0.04 to 0.10)	39	0.12 (0.08 to 0.16)	46	0.14 (0.10 to 0.19)	69	0.22 (0.17 to 0.27)
≥ 80 yrs	13,231	9	0.07 (0.02 to 0.11)	13	0.10 (0.05 to 0.15)	18	0.14 (0.07 to 0.20)	31	0.24 (0.16 to 0.33)

### Model development, training, and validation

**Fig. 1:**
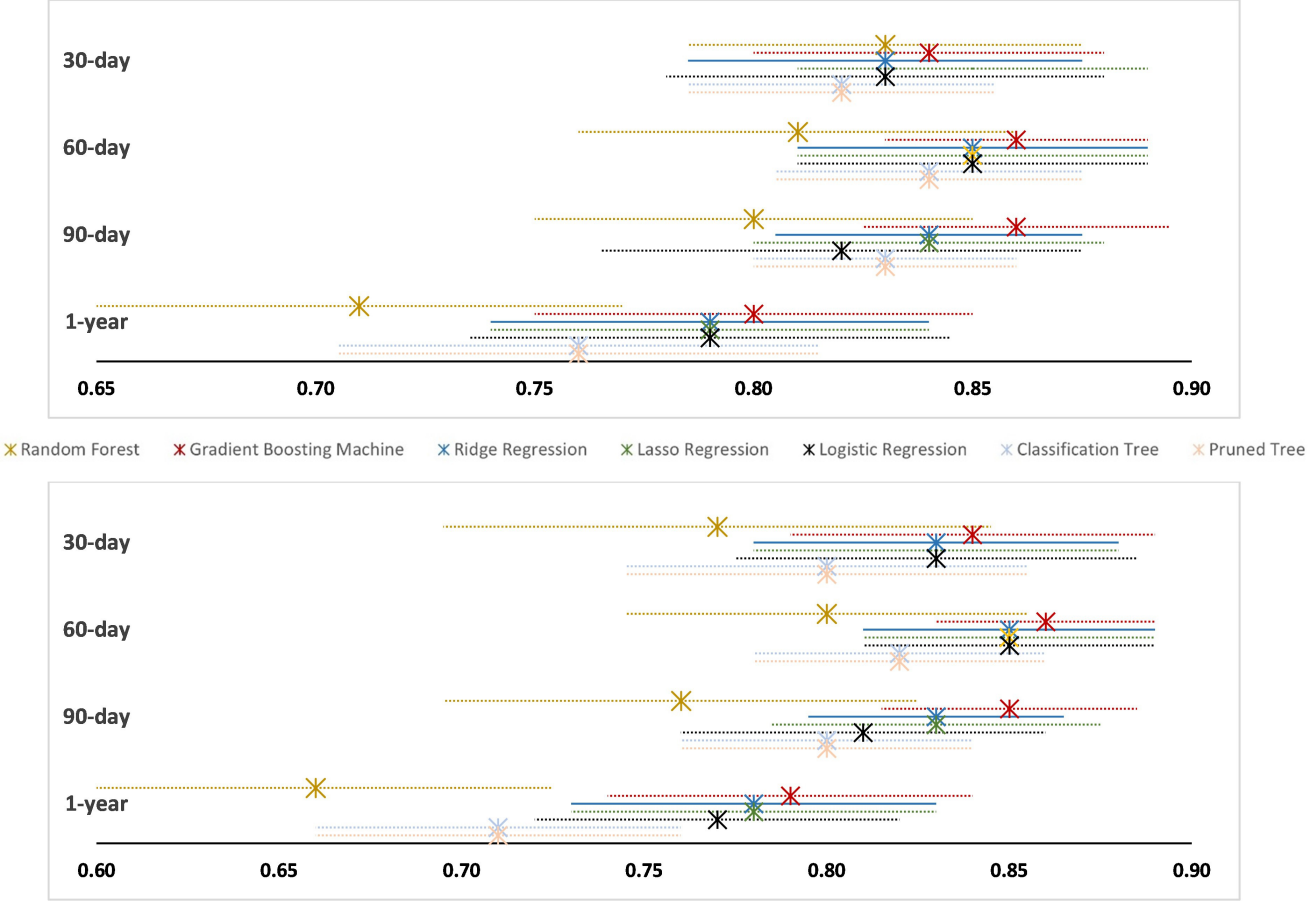
Forest plot of implant failure predictive models’ area under the curve (AUC) values in the Swedish Arthroplasty Register; revision (top) and reoperation (bottom). Dotted lines represent the CI.

Gradient boosting machine (GBM) was consistently the top-performing ML algorithm in predicting revision and reoperation risks due to PPFF at all timepoints, except for 30-day revision, in which least absolute shrinkage and selection operator (LASSO) regression outperformed GBM by a narrow margin. Prediction performance of the applied ML algorithms in the internal validation cohort are presented in Table V. All models exhibited a remarkably similar AUC of approximately 0.80 (Table V, Figure 1).

**Table V.** Implant failure predictive model performance in the Swedish Arthroplasty Register.

Outcome	Model performance: AUC in % (95% CI)						
	**Random forest**	**Gradient boosting machine**	**Ridge regression**	**Lasso regression**	**Logistic regression**	**Classification tree**	**Classification tree with pruning**
**Revision due to PPFF**							
30 days	0.83 (0.79 to 0.88)	0.84 (0.80 to 0.88)	0.83 (0.79 to 0.88)	0.85 (0.81 to 0.89)*	0.83 (0.78 to 0.88)	0.82 (0.78 to 0.85)	0.82 (0.78 to 0.85)
60 days	0.81 (0.76 to 0.86)	0.86 (0.83 to 0.89)*	0.85 (0.81 to 0.89)	0.85 (0.81 to 0.89)	0.85 (0.81 to 0.89)	0.84 (0.80 to 0.87)	0.84 (0.80 to 0.87)
90 days	0.80 (0.75 to 0.85)	0.86 (0.82 to 0.89)*	0.84 (0.81 to 0.88)	0.84 (0.80 to 0.88)	0.82 (0.76 to 0.87)	0.83 (0.80 to 0.86)	0.83 (0.80 to 0.86)
1 year	0.71 (0.65 to 0.77)	0.80 (0.75 to 0.85)*	0.79 (0.74 to 0.84)	0.79 (0.74 to 0.84)	0.79 (0.73 to 0.84)	0.76 (0.71 to 0.82)	0.76 (0.71 to 0.82)
**Reoperation due to PPFF**							
30 days	0.77 (0.69 to 0.84)	0.84 (0.79 to 0.89)*	0.83 (0.78 to 0.88)	0.83 (0.78 to 0.88)	0.83 (0.77 to 0.88)	0.80 (0.74 to 0.85)	0.80 (0.74 to 0.85)
60 days	0.80 (0.75 to 0.86)	0.86 (0.83 to 0.89)*	0.85 (0.81 to 0.89)	0.85 (0.81 to 0.89)	0.85 (0.81 to 0.89)	0.82 (0.78 to 0.86)	0.82 (0.78 to 0.86)
90 days	0.76 (0.69 to 0.82)	0.85 (0.81 to 0.88)*	0.83 (0.80 to 0.87)	0.83 (0.78 to 0.87)	0.81 (0.76 to 0.86)	0.80 (0.76 to 0.84)	0.80 (0.76 to 0.84)
1 year	0.66 (0.60 to 0.73)	0.79 (0.74 to 0.84)*	0.78 (0.73 to 0.83)	0.78 (0.73 to 0.83)	0.77 (0.72 to 0.82)	0.71 (0.66 to 0.76)	0.71 (0.66 to 0.76)

* Best-performing model for each outcome.

AUC, area under the receiver operating characteristic curve; PPFF, periprosthetic femoral fracture.

### Feature importance and response analysis

Lasso regression achieved the highest AUC value for 30-day revision prediction while GBM was the top-performing model for the all-outcome endpoints. The following sub-sections delve into the analysis of response and feature importance. While these features provide insights into the model internal decision-making process, caution is strongly advised when interpreting the results, since feature importance is not subject to inferential testing and does not establish statistical significance.

### 30-day periprosthetic femoral fracture

**Fig. 2:**
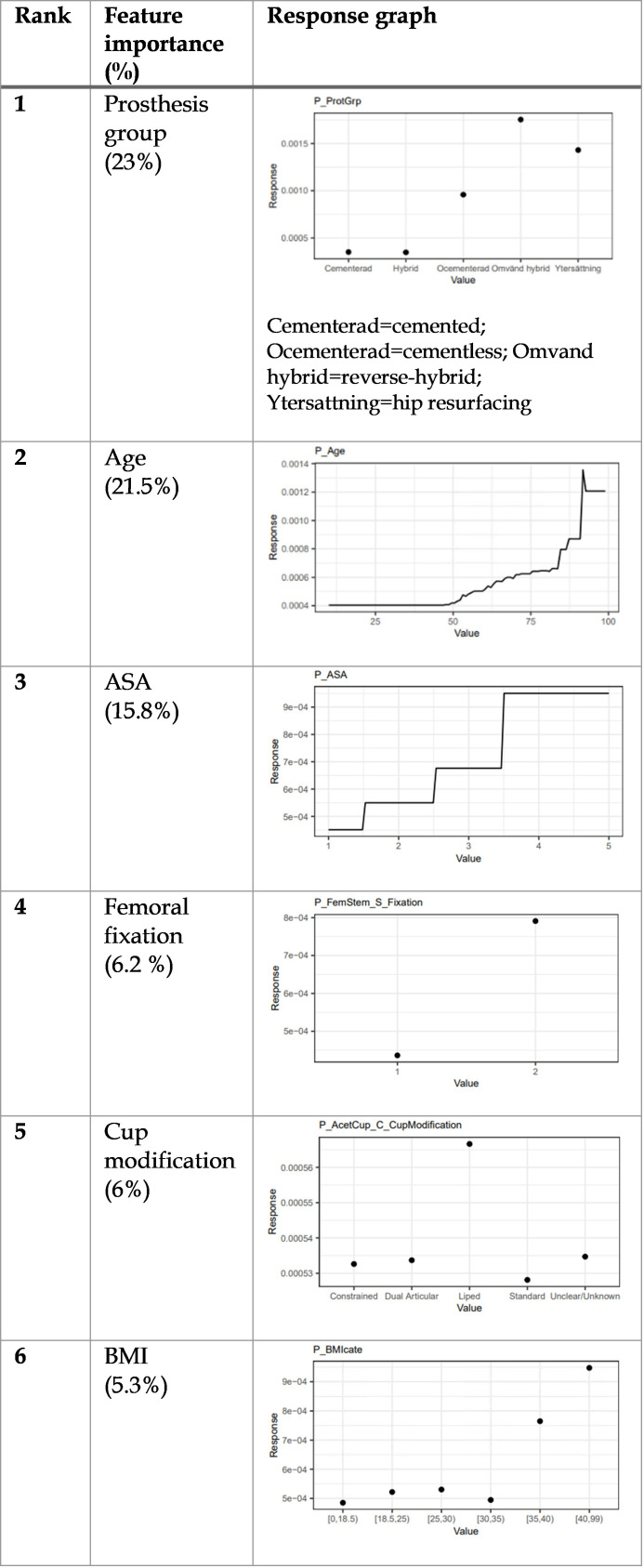
The most important features for the gradient boosting machine 30-day reoperation predictive model. ASA, American Society of Anesthesiologists.

Lasso regression and GBM were the best-performing ML models for 30-day revision and reoperation due to PPFF, respectively. Table VI and Figure 2 identified the key features driving these predictions. The choice of prosthesis, primarily cementless femoral fixation or hip resurfacing, increased fracture risk leading to revision or reoperation, while patient features – namely obesity class III and higher ASA grades – played a secondary key part. Age and flanged cups were both identified as drivers for increased reoperation risk due to femoral fracture but not revision, which had an increased risk with the use of monoblock stems.

**Table VI.** The most important features for the Lasso regression 30-day revision predictive model.

Rank	Feature	Coeff.
1	Cementless femoral fixation	+ 2.93
2	Femoral monoblock	+ 2.40
3	Resurfacing	+ 1.04
4	Idiopathic necrosis	+ 0.73
5	WHO obesity class III	+ 0.67
6	ASA grade	+ 0.48
7	Reverse hybrid fixation	+ 0.41

ASA, American Society of Anesthesiologists; WHO, World Health Organization.

### 60-day periprosthetic femoral fracture

**Fig. 3:**
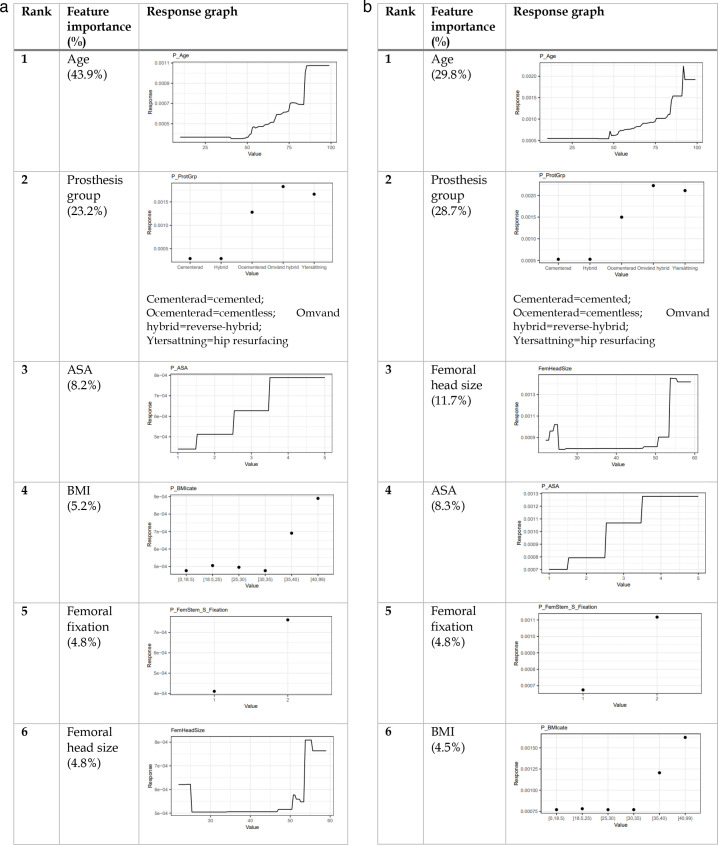
The most important features for the gradient boosting machine 60-day implant failure predictive models; revision (left) and reoperation (right). ASA, American Society of Anesthesiologists.

Increasing age, BMI, and ASA grade variably seemed to increase the incidences of PPFF leading to revision or reoperation (Figure 3). Like the 30-day outcome, cementless femoral fixation, hip resurfacing, and reverse hybrid fixation increased fracture risks necessitating an intervention. Large femoral head sizes (> 45 mm) or very small ones (< 25 mm) appeared to be a relatively important feature used in the GBM models, accounting for approximately 5% and 12% of the predictive power for revision and reoperation, respectively.

### 90-day periprosthetic femoral fracture

**Fig. 4:**
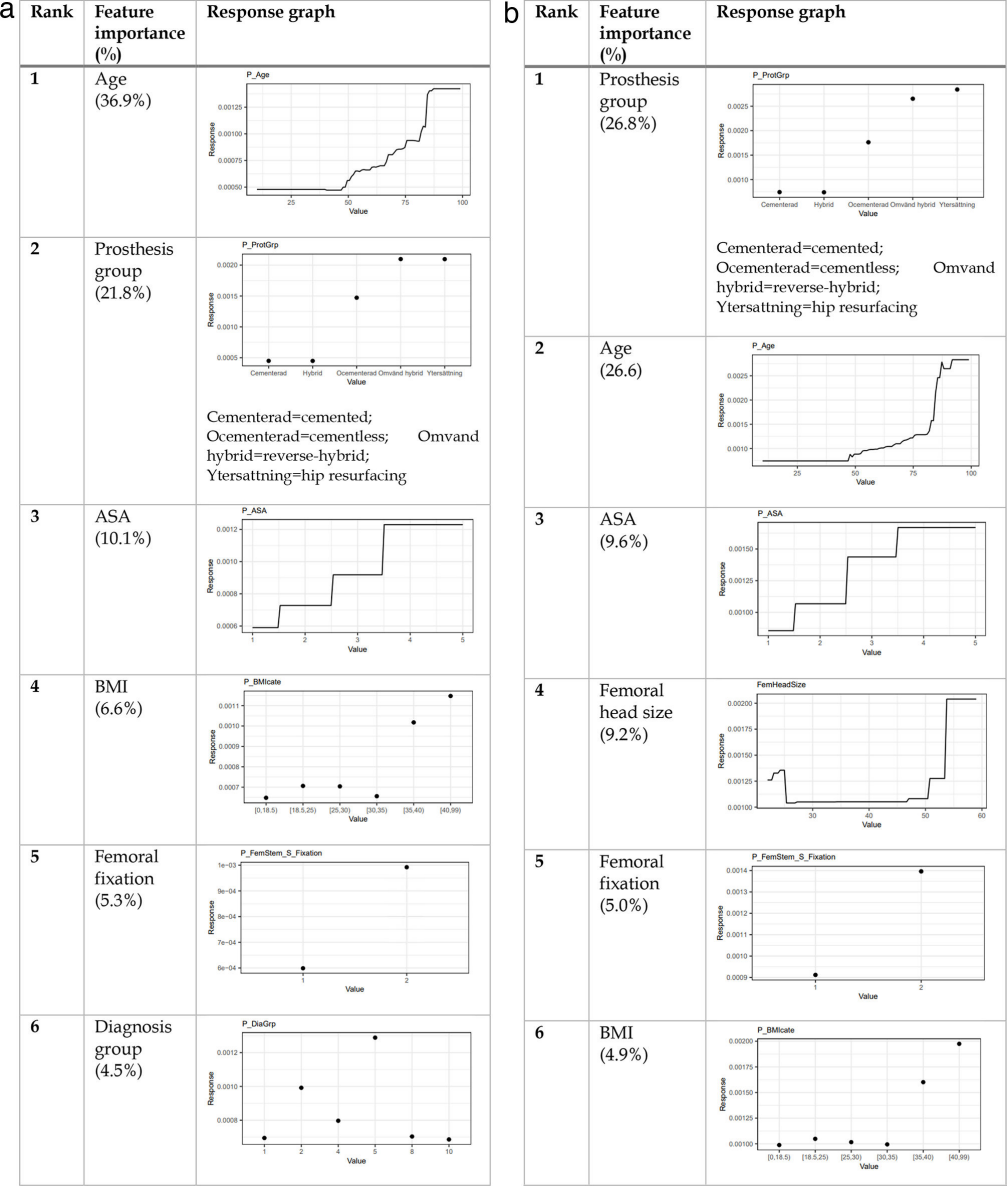
The most important features for the gradient boosting machine 90-day implant failure predictive models; revision (left) and reoperation (right). ASA, American Society of Anesthesiologists.

When compared with the 60-day fracture, no significant changes in response graphs were observed for the 90-day endpoint (Figure 4). In the revision model, diagnosis group (idiopathic necrosis), rather than femoral head size, was ranked in the top six features.

### One-year periprosthetic femoral fracture

**Fig. 5:**
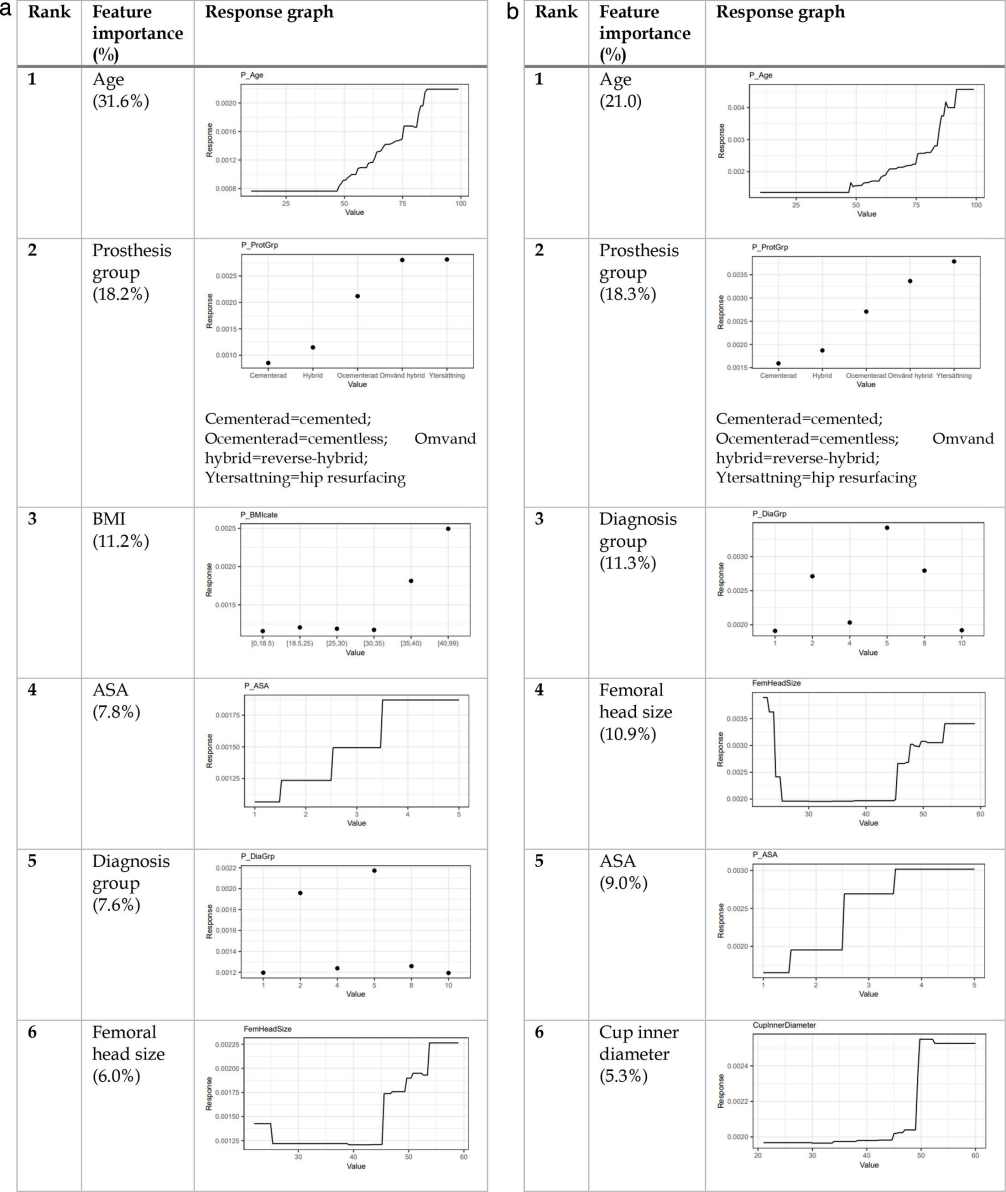
The most important features for the gradient boosting machine one-year implant failure predictive models; revision (left) and reoperation (right). ASA, American Society of Anesthesiologists.

At the one-year interval, preoperative diagnosis of idiopathic necrosis or inflammatory arthritis became apparent and increased long-term secondary surgery risk (Figure 5). Similar observations were found regarding very small (< 25 mm) or large (> 45 mm) femoral head sizes. Within the prosthesis group, hip resurfacing was assigned a slightly higher weight compared to fixation type.

## Discussion

This study has demonstrated that ML-based predictive models for revision and reoperation due to PPFF after primary hip arthroplasty achieved a relatively higher level of accuracy, as determined by the AUC, compared to the logistic regression linear model. To our knowledge, this is the first study to develop individualized risk assessment for PPFF.

Descriptive statistics of the SAR database revealed no statistically significant difference temporal trends concerning the cumulative incidences of both revision and reoperation due to femoral fracture, suggesting consistency in these measures over the studied time period. While no sex discrepancies were apparent, the incidence of fractures seemed to be most pronounced in the 50- to 79-year age groups. Although the recent SAR annual report lacks specific data related to PPFF, it highlights that the number of revisions performed in the past decade was relatively constant with no significant differences based on sex.^7^ In contrast, the 2022 report from the National Joint Registry (NJR), which uses data from England, Wales, Northern Ireland, the Isle of Man, and the States of Guernsey, highlighted a decline in the incidence of revisions in the last decade compared to the year 2008, and an inverse relationship between revision probability and the patient’s age with greater variability among females at younger ages and among males at older ages.^9^ A New Zealand Joint Registry study showed a markedly greater lifetime risk of PPF following primary THA in males across all age bands, and particularly in the younger age groups (5.31%:2.82% in the 45- to 50-year age band).^23^

Our ML models showed good binary discriminative power in distinguishing between the positive and negative cases across various threshold settings, ranging between AUC 0.79 and 0.86 for both revision and reoperation predictions due to femoral fractures. The GBM was consistently the best performing model (except for 30-day revision; GBM AUC 0.84, LASSO AUC 0.85), and marginally outperformed the linear logistic regression model. Our model has a practical application in surgical practice and can be implemented either using a formula or a web calculator interface.

Two studies developed predictive models to forecast the risks of revision and re-revision following primary THA.^24,25^ Klemt et al^24^ were able to achieve a discriminative ability of AUCs of 0.82 to 0.87 for all-case revision and 0.80 to 0.86 for re-revision following THA.^25^ However, their models were developed on data derived from a single tertiary referral centre in the USA, had a limited sample size (revision = 566, re-revision = 408), and did not include disease severity, preoperative diagnosis, or implant-specific factors in their models – all of which were observed to be leading players in driving our predictions.

Our findings identified several patient characteristics and implant-specific features to be associated with revision and reoperation after THA due to PPFF at each timepoint. For the early (30 to 90 days) secondary surgery, these included: 1) patient features (increasing age and comorbidities (ASA grade > III), obesity classes II and III, and higher ASA grade (> III) increased risks of both revision and reoperation); 2) preoperative diagnosis (idiopathic necrosis increased revision risk); and 3) implant design (cementless femoral fixation, reverse hybrid fixation, hip resurfacing, and small (< 25 mm) or large (> 45 mm) femoral heads increased both risks).

In addition to the abovementioned characteristics, a preoperative diagnosis of inflammatory arthritis was identified as having increased revision and reoperation risks at one year.

The risk factors identified in this study are in concordance with previous observational studies.^26^ Prokopetz et al’s^26^ systematic review of 86 studies found younger age, greater comorbidity, preoperative diagnosis of avascular necrosis, and the extremes of femoral head size all to be associated with increased revision risk. However, their findings with regard to prosthesis materials and fixation method, while favouring the use of cement, were inconclusive, and this could be attributed primarily to the methodology employed, which excluded studies of small sample sizes and those examining the failure of a specific component, such as the stem. In contrast to the available evidence suggesting that youth predisposes patients to exceeding the lifetime risk of revision surgery,^27,28^ our study focused on a specific reason for short-term (up to one year) secondary surgery due to femoral fracture; thus, our augmented one-year implant failure risk with increasing age is likely attributed to the underlying physiological processes of ageing.^29,30^ With regard to comorbid status and BMI, our findings support the available evidence indicating increased odds of early revision THA with higher comorbidity indices^31,32^ and BMI.^33-37^ Our study showed that prosthesis choice, specifically cementless femoral fixation and hip resurfacing arthroplasty, seems to be the strongest risk factor for PPFF. That said, the limitations of using revision as an endpoint outcome when making comparisons between implants ought to be considered.

### Strengths and limitations

The study findings should be interpreted in light of several limitations. First of all, the limitations related to the retrospective analysis of prospectively collected observational data in a single national joint registry, as well as the limitations concerning the use of revision/reoperation as endpoint outcomes, may hinder the clinical applicability to other settings. The large amount of missing data, particularly incomplete ASA grades in SAR prior to 2008, and our assumption of missing data being completely at random by eliminating missing observations rather than imputing variables, may have introduced bias to the findings. However, imputation introduces uncertainty and may result in biased estimates, which our methodology aimed to avoid.^38^ Similarly, our models were compared using the AUC metric, which is regarded as a gold standard in evaluating classifier performance across a range of thresholds and allows comparisons of multiple ML models. Nonetheless, the AUC-ROC curve-based metric neglects to address the balance of true positives and true negatives, and the potential bias introduced by the low incidence of reoperation, which may affect the accuracy and generalizability of the predictive model which alternative measures seek to address. However, the need to specify a threshold in other approaches is known to introduce variations in clinical practice. The choice of λ in the LASSO model involves a trade-off between bias and variance, potentially excluding important features if too large or retaining irrelevant ones if too small, although cross-validation was used for validation. Additionally, the feature importance identified by our models, while seeking to provide insights into the risk factors, are not subject to inferential testing assumptions, and hence cannot establish associations. The use of discrete timepoints rather than survival analysis limits the study’s ability to fully assess temporal variations in fracture incidence and beyond one year postoperatively. Moreover, our study investigated predefined outcomes but did not address PPFs that progress to fixation rather than revision; further studies are required to investigate this area. Future studies should aim to externally validate our ML models on other datasets, such as the NJR in the UK, and prospective clinical trials are required to investigate the effects of certain risk factors like comorbidity indices and fixation methods on patient outcomes.

This is the first study to develop ML models to forecast the risk of PPFFs necessitating secondary surgery. Several risk factors were identified by our models: cementless fixation type; hip resurfacing arthroplasty; the extremes of femoral head sizes; a preoperative diagnosis of idiopathic necrosis or inflammatory arthritis; and increasing age, BMI, and ASA comorbidity grades. Our validated ML models on a large-scale national database showed good discrimination that estimates individuals’ risk of femoral fracture and can be readily applied to surgical practice to aid clinical decision-making.

## Data Availability

The data that support the findings for this study are available to other researchers from the Swedish Arthroplasty Register steering committee upon reasonable request.
